# Annotating and indexing scientific articles with rare diseases

**DOI:** 10.1186/s13326-025-00346-1

**Published:** 2026-01-06

**Authors:** Hosein Azarbonyad, Zubair Afzal, Rik Iping, Max Dumoulin, Ilse Nederveen, Jiangtao Yu, Georgios Tsatsaronis

**Affiliations:** 1 Elsevier B.V., Amsterdam, Noord Holland The Netherlands; 2 Erasmus MC, Rotterdam, Zuid Holland The Netherlands

**Keywords:** Annotation, Indexing, Rare diseases, Scientometrics, Bibliographic databases, Natural language processing, Health sciences, Research applications

## Abstract

**Background:**

Around 30 million people in Europe are affected by a rare (or orphan) disease, defined as a condition occurring in fewer than 1 in 2,000 individuals. The primary challenge is to automatically and efficiently identify scientific articles and guidelines that address a particular rare disease. We present a novel methodology to annotate and index scientific text with taxonomical concepts describing rare diseases from the OrphaNet taxonomy. This task is complicated by several technical challenges, including the lack of sufficiently large, human-annotated datasets for supervised training and the polysemy/synonymy and surface-form variation of rare disease names, which can hinder any annotation engine.

**Results:**

We introduce a framework that operationalizes OrphaNet for large-scale literature annotation by integrating the TERMite engine with curated synonym expansion, label normalization (including deprecated/renamed concepts), and fuzzy matching. On benchmark datasets, the approach achieves **precision = 92%**, **recall = 75%**, and **F1 = 83%**, outperforming an string-matching baseline. Applying the pipeline to Scopus produces disease-specific corpora suitable for bibliometric and scientometric analyses (e.g., institution, country, and subject-area profiles). These outputs power the *Rare Diseases Monitor* dashboard for exploring national and global research activity.

**Conclusion:**

To our knowledge, this is the first systematic, scalable semantic framework for annotating and indexing rare disease literature at scale. By operationalizing OrphaNet in an automated, reproducible pipeline and addressing data scarcity and lexical variability, the work advances biomedical semantics for rare diseases and enables disease-centric monitoring, evaluation, and discovery across the research landscape.

## Introduction

In Europe, there is a population of 30 million people enduring the challenges of a rare (or orphan) disease. A disease or syndrome is considered to be rare if it affects fewer than 1 in $2,000$ people or if it is a tumor which occurs in less than 6 in $100,000$ people per year. Only in the Netherlands this accounts to 6–8% of the population^1^. There are over $8,000$ rare diseases known, and new rare diseases are identified regularly. Because of the low incidence, expertise is often limited and most patients can only go to one or two locations to receive the care they need, if such a location with this expertise is at all available. Due to the high degree of specialization required, the care and research for patients with rare diseases are predominantly centralized within specialized organizations, such as University Medical Centers (UMCs) in the Netherlands. The Dutch UMCs consider this care and research a great responsibility in order to improve the lives of patients with a rare disease. In order to increase quality standards and to gain more insight for patients where true expertise is present and to make membership of the European Reference Networks (ERN)^2^ possible, the Dutch government has put in place a system of national acknowledgment for centres of expertise on rare diseases. To become an expert centre an institution has to formally apply and provide evidence of patient care and research activities. Acknowledgment as an expert centre is an important boost for the viability and continuity of care and research, and is a stepping stone towards becoming part of larger structures for rare diseases in Europe (ERN). Similar initiatives around the world have commenced to both recognize expertise around the rare diseases, and facilitate and support advancements on controlling or treating such diseases.

A (candidate) centre of expertise is expected to conduct (fundamental) scientific research in the field of the rare disorder(s) for which, acknowledgment has been requested, and to publish about the results. Insights into research on rare diseases are therefore relevant. However, an easy way of identifying research activity in rare diseases is lacking. There is no readily available classification scheme available in any of the publication databases. Knowing research activity on a rare disease and comparing this to other medical centres can be useful to:

- Connect researchers and clinicians from different disciplines and throughout fundamental, transnational and clinical research.
- Identify and promote collaboration with other institutions working on similar topics.
- Give patients and their representatives further insight in where and what kind of research is conducted.
- Identify unique activity on certain rare diseases for which applying for acknowledgement (by ERN) can be very promising and meaningful.
- Provide evidence and numbers to support applications.

An effective approach to quantify and validate research pertaining to diverse rare diseases involves conducting a comprehensive bibliometric analysis of scientific publications. In this study, we propose a novel framework for annotating and indexing research articles related to rare diseases, utilizing the OrphaNet taxonomy  [1], the most extensive classification system capturing relationships between diseases, genes, and other relevant features. OrphaNet stands as the largest repository of rare disease taxonomy, providing a robust foundation for this research. Through the indexing of articles based on concepts from this taxonomy, we can systematically monitor and evaluate distinct research activities associated with each specific disease. This approach not only enables a deeper understanding of the current state of research but also facilitates tracking advancements and trends in the realm of rare diseases. By embracing this framework, we can gain valuable insights into the scientific landscape and foster greater collaboration and progress within the field.

To conduct an in-depth analysis of rare diseases, we primarily focus on articles published within the last ten years, extracting their abstracts, titles, and keywords from the Scopus^3^ database. Since there is no sufficiently large dataset to train a supervised model for indexing, we opt for unsupervised methods. Among the available unsupervised approaches for text indexing with concepts from a taxonomy, we select TERMite^4^, a highly scalable named entity recognition tool known for its extensive coverage of the OrphaNet taxonomy. Our pipeline begins with article pre-processing, followed by utilizing TERMite to match the concepts in the OrphaNet taxonomy to the articles. This matching process relies on both exact and fuzzy matching of disease names and their synonyms within the text. It is essential to mention that although TERMite offers a substantial coverage of the OrphaNet taxonomy, it does not cover the entire taxonomy due to its evolutionary nature (concepts can be added/removed to the taxonomy). To overcome this limitation, we enhance the predictions by combining TERMite’s annotations with a straightforward exact matching approach, wherein we perform a dictionary lookup of disease names (and their synonyms) within the text. Using this approach, we can efficiently and comprehensively index a large volume of articles.

We conducted an evaluation using a set of publicly available datasets to assess the performance of our designed system in indexing articles related to rare diseases. The obtained results demonstrate that our proposed system exhibits high precision and recall in effectively indexing such articles. Moreover, we carried out a user-study to assess the quality of assigned OrphaNet concepts to articles published by four major medical centers in the Netherlands. The findings from this study confirm the superiority of our TERMite-based approach when compared to an exact matching baseline. Our research reveals a staggering 2.4 million articles published globally within the past ten years on the subject of rare diseases. Utilizing these extensive annotations, we have developed the *‘Rare Diseases Monitor’*^5^ dashboard, a powerful tool for monitoring advancements in the field of rare diseases, specifically within Dutch publications. The successful outcomes of our study underscore the efficacy and relevance of our system and provide valuable insights into the ever-evolving landscape of rare disease research.

Beyond applying an existing named-entity recognition engine, this study contributes to the field of biomedical semantics by establishing the first large-scale semantic framework for annotating and indexing rare disease literature. We integrate the OrphaNet taxonomy into an automated pipeline, creating precise mappings between ontology concepts and textual mentions. This process required handling synonymy, polysemy, and lexical variation unique to rare disease nomenclature. The resulting semantic layer enables consistent concept-level indexing across millions of scientific articles, forming the basis for large-scale bibliometric and scientometric analyses. In doing so, our work extends the scope of biomedical semantics to a domain that has, until now, lacked a systematic semantic infrastructure.

The remainder of this paper is organized as follows. Section 2 discusses the related work to the current study. In Sect. 3, we describe the details of the system used for indexing articles with OrphaNet concepts. In Sect. 4, the details of the datasets and resources used to build and evaluate the proposed system is discussed. Section 5 contains the details of the experiments and their results around building and evaluating different systems. Section 6 discusses the main findings, the limitations, and the future work. Finally Sec. 7 concludes the paper and discusses the limitation of the current study and possible future work.

## Related work

We review the related work from two angles: classification of documents using taxonomies, and the research on building solutions around automatically annotating documents with biomedical taxonomies.

In a broader context, mapping documents into taxonomies can be seen as a document classification task. Document classification is the task of classifying text documents to one or multiple labels  [2]. The range of approaches for the standard text classification task varies from the traditional feature engineering based methods  [3–5] to the advanced deep learning approaches  [6–8]. The most common theme among most of these approaches is the need for a set of manually annotated documents from which these models can learn the associations between labels and documents. A specific kind of document classification task is the hierarchical document classification in which labels themselves are organized into a semantic hierarchy  [9]. Examples of such hierarchies are taxonomies such as MeSH  [10] or OrphaNet  [11] which not only contain the concepts (labels) but also contain the relation between them. Several strong neural architectures such as convolutional neural networks  [12, 13], recurrent neural networks  [12, 14], taxonomy embedding methods  [15], and recently transformers-based models  [16, 17] have been successfully used to train hierarchical text classification models on several datasets. Despite having a strong performance, most (if not all) of these models require a large amount of training (labeled) samples to train an effective model in a supervised setting. There have been some efforts on modeling the task as a semi-supervised learning task  [18, 19] in which a large set of unlabeled documents are used to augment a relatively small training set. While these methods help reduce the workload of human annotation, they still rely on a labeled dataset that encompasses all classes. However, acquiring such a dataset can be expensive, especially when dealing with a taxonomy containing numerous concepts. To overcome this issue, several approaches looked into the possibility of performing unsupervised hierarchical text classification in which there is no need for a labeled set of documents  [20, 21]. Traditional approaches on unsupervised hierarchical text classification heavily rely on term statistics or exact matching on terms in text which might be problematic especially when there is no known corpus related to the taxonomy of interest or when there is synonymy and polysemy in the taxonomy. Recent unsupervised approaches use methods such as word embeddings to transform both documents and concepts into a high-dimensional space and then estimate their similarities  [22]. Such approaches do not generalize well for taxonomies with very specific terms (such as OrphaNet) as such terms do not occur in the vocabulary of the embedding models trained usually on generic web/book content.

Indexing documents with biomedical taxonomies such as MeSH, ICD10  [23], UMLS  [24], and MedDRA  [25] is an active research area due to its high impact and numerous applications  [26–29]. MeSH is a widely-used taxonomy for this task due to its size and high coverage across different disciplines. Ranking-based approaches are the most used methods for indexing documents with MeSH terms  [28] mainly due to the large number of labels in the taxonomy which hinders scalibilty of traditional text classification models on this taxonomy. The main idea behind the ranking-based approach is to use a set of annotated documents as the reference points. For a new unlabeled document, first the similarity of the document with the labeled ones is calculated, then the labels from the most similar documents are aggregated and the top ones are used as the label for the new document. The idea is further extended by using more advance learning to rank models which instead of a similarity metric (or a classifier) try to directly learn a function that can rank labels given a document  [30]. Deep learning approaches have also been used on top of datasets with MeSH terms  [12]. It is worth noting that all these approaches require a set of annotated documents. Similar to MeSH, methods for indexing documents with ICD10 or other taxonomies concepts are rule-based  [31], traditional machine learning models  [32], or deep learning models  [33]. The main issue with most of these approaches is their need to a set of annotated documents. To the best of our knowledge, there is no system or model designed for indexing documents with OrphaNet concepts. None of the above approaches are directly applicable to the OrphaNet taxonomy.

## Methods

**Fig. 1:**
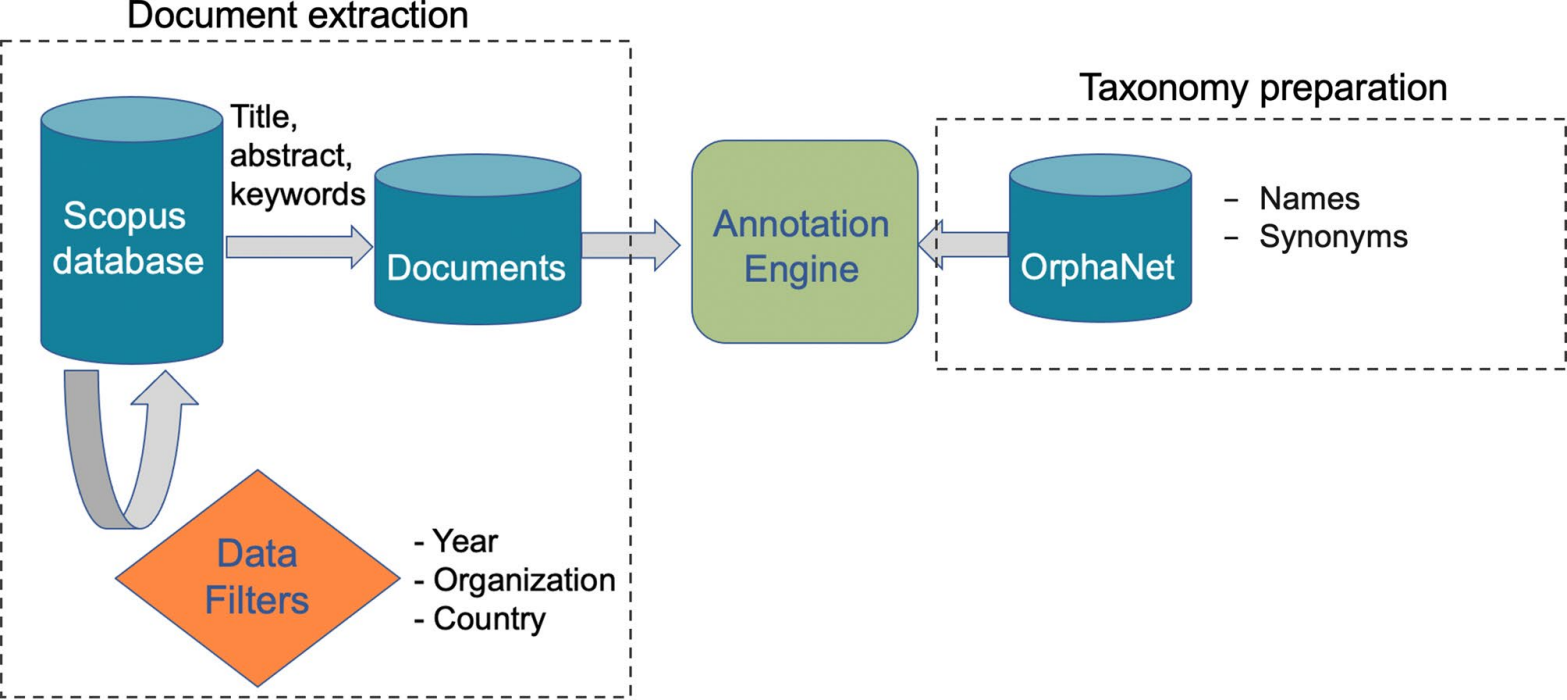
An overview of the pipeline for annotating articles with rare diseases

Our goal is to match research outputs (articles and books) to rare diseases. The rare diseases of interest are coming from the OrphaNet taxonomy which has a hierarchical structure (top level nodes are generic diseases and the leaves are the most specific diseases). Therefore, this task can be seen as a hierarchical text classification task where the goal is to classify a given text (article) as being relevant to one or multiple rare diseases. Since there is no manually labeled dataset with rare diseases, we treat this task as an unsupervised text classification task where we try to match articles and diseases based on the textual similarity of articles and textual information available for diseases in the rare diseases’ taxonomy. The general pipeline for indexing articles with rare diseases in shown in Fig. 1.

The main components of this pipeline are document extraction, taxonomy preparation, and the annotation engine. In the remainder of this section, we describe the details of these components.

### Document extraction and preparation

The very first step of the pipeline is building a collection of articles to be matched against the taxonomy. Scopus is one of the largest publication databases containing about 80 million unique research outputs. Each record in this database corresponds to an article and contains some textual information such as abstract and keywords as well as other types of metadata such as the affiliation of the authors and publication year. This metadata can be used to perform several interesting analyses once the matching is done such as tracking the most active organizations on different rare diseases. Therefore, we choose to use this database containing the rich set of metadata around articles. We use the articles published in Scopus in the past ten years (2012–2022). This subset contains over 36 million articles which are used to build the search corpus. A document in our search corpus corresponds to an article. To build a document, we concatenate the title, abstract, and keywords of the corresponding article and separate these fields with a special token ([SEP]). We only use these fields as they are publicly available and based on our experiments, using full text of articles leads to a lot of noise (mainly false positives). We perform some simple text pre-processings on the documents including punctuation and non-alphanumeric character removal. Then, we feed these documents to the annotation engine which matches them to the rare diseases.

### Taxonomy preparation

As rare diseases taxonomy, we use OrphaNet which is a structured vocabulary for rare diseases capturing relationships between diseases, genes and other relevant features as well as some basic information around the disease itself such as the name and its synonyms. This taxonomy is created by French National Institute for Health and Medical Research in 1997. The current taxonomy contains 9,287 concepts (diseases) organized in a hierarchy. This taxonomy is updated monthly and the standard guidelines are followed for deprecation of concepts. Due to the dynamic nature of this taxonomy, any indexing engine built on top of this taxonomy should be able to adapt for such changes. This requires the annotation engine to be computationally fast and flexible. As mentioned, there is no labeled dataset with rare diseases labels, therefore, we consider the task an unsupervised classification task. For matching diseases to the documents, we use the textual names of diseases as well as their synonyms. Any detection of a disease name or its synonyms in a document is considered as a match for the disease.

### Annotation engine

Annotation engine is the core component of the annotation pipeline which does the matching between documents and diseases. The documents and diseases are the two inputs of this engine. We have experimented with several existing annotation tools to perform the matching. Most of these tools are designed to annotate documents with not exactly the OrphaNet but taxonomies related to it such as MeSH. The main drawback of these tools is their low coverage to the OrphaNet (see Table 1).

**Table 1:** Coverage of existing bio-medical taxonomies for OrphaNet

Taxonomy	Coverage
MeSH	0.11
ICD10	0.46
UMLS	0.31
MedDRA	0.08

**Fig. 2:**
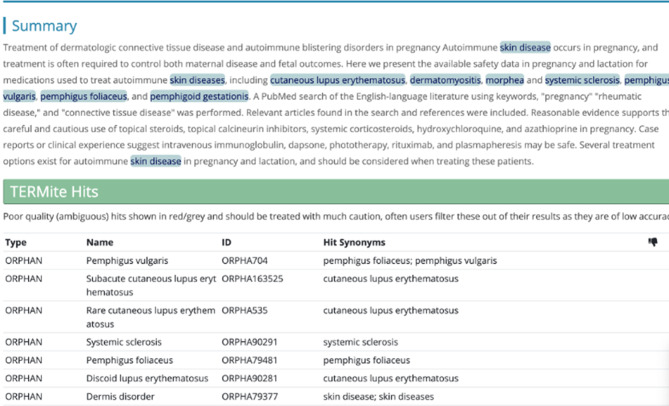
An example annotation of the TERMite engine on a given document

SciBite’s TerMite is the only annotation engine with a very high coverage for the OrphaNet (it covers 98% of OrphaNet concepts). Therefore, we use this tool as our main annotation engine. TerMite is an NER tool that rapidly scans and semantically annotates raw text (up to 1 million words per second) with entities from over 50 biopharma and biomedical taxonomies. OrphaNet is one of the taxonomies covered in this tool. Figure 2 depicts an example annotation done by this engine on a document.

For matching documents to diseases, TERMite uses a string matching and a fuzzy matching approach. TERMite is essentially a named entity recognition tool that employs a set of techniques to identify and extract named entities from text data. TERMite utilizes a combination of rule-based and statistical methods to achieve high precision and recall rates in entity recognition. The tool adopts a two-step approach, beginning with a very simple pre-processing phase where text data is tokenized and normalized. In the second step of TERMite, a rule-based matching mechanism is employed to identify named entities based on predefined patterns and linguistic rules. These rules encompass a wide range of entity types, but for our usecase, we only use the OrphaNet as the underlying taxonomy. The rule-based approach allows for precise identification of entities that conform to specific patterns, such as capitalization, word combinations, or context-specific cues. Since disease names are usually very specific, this approach is very well-suited for our usecase. Additionally, to increase the recall and handle variations or inconsistencies in named entities that may arise due to misspellings, abbreviations, or slight modifications, we use the fuzzy matching capabilities within TERMite. Fuzzy matching allows TERMite to identify entities that are similar to the ones specified in the predefined rules or statistical models, even if they are not an exact match. TERMite’s fuzzy matching was used with its default similarity threshold (score ≥ 0.8) for token-based string matching. The OrphaNet terminology within TERMite is regularly synchronized with public releases.

As mentioned, TERMite covers 98% of the OrphaNet. The remaining 2% is not covered mainly due to the recent changes to the taxonomy which is not captured by TERMite. To cover the remaining 2%, we perform a simple string matching based on the name and synonyms of diseases which are not covered by TERMite. TERMite does not take the hierarchical structure of the concepts into account. To take the hierarchy into account, all articles matched with a concept are propagated and assigned to the ancestor concepts (with distance less than three concepts to the concept) in the taxonomy tree.

## Datasets and resources

As article collection we use the articles in the Scopus published in the past ten years (2012–2022) containing over 36 million records. We use title, abstract, and keywords of articles as well as the publication year and affiliation for performing different analyses and filtering on the data. We use disease names and synonyms (as described in Sect. 3) to perform the matching.

Since there is not any similar methodology to index articles (or any text) with rare diseases and there is no labelled dataset to train and evaluate supervised models for this task, we choose the string-matching as the baseline and compare its performance to the TERMite-based annotation pipeline. The string-matching approach matches disease names and their synonyms in documents. We evaluate the performance of the proposed by means of a set of publicly available datasets (Sect. 4.1) and a user study (Sect. 4.2).

### Datasets for evaluation

There is no large publicly available text collection with OrphaNet terms assigned to them. Therefore, we use a set of publicly available dataset annotated with MeSH terms to evaluate the performance of the annotation engine. To evaluate the performance of our approach, we filter these datasets and only keep samples which have a MeSH term that is also present in the OrphaNET taxonomy. By doing this, we can directly use the resulting filtered dataset for evaluation of our rare diseases annotation pipeline as this dataset will only contain OrphaNet terms assigned to documents. We compile a dataset by combining three datasets:

- **NLM2007** [28] which contains 200 PubMed documents (titles and abstracts) created in the NLM2007 initiative. After filtering samples (documents) containing OrphaNet terms, we end up with 11 samples from this dataset.
- **BioASQ5000**  [34] which contains 5000 PubMed documents (title and abstracts) annotated with MeSH terms. This dataset was developed as part of the BioASQ (Biological Answering Systems) challenge, which aims to advance the state of the art in biomedical question answering. The resulting dataset contain 223 documents, after performing the filtering.
- **L1000**^6^ dataset is a comprehensive collection of gene expression data generated through the LINCS program. It captures the changes in gene expression levels in response to various small molecule perturbations. This dataset contains 1000 samples annotated with MeSH terms. After filtering, we end up with 45 samples annotated with OrphaNet terms.

In total, by the filtering done on the above datasets, we collected a dataset with 279 documents annotated with OrphaNet concepts. This dataset covers 352 unique rare diseases. We use this annotated dataset to evaluate the performance of our approaches in indexing documents with rare diseases concepts.

### Dataset for the user study

For further evaluation of the annotation pipeline, we build a small evaluation experiment in which we use the annotation pipeline to assign diseases to the articles. Then, to build a manageable user study experiment, we focus on four UMCs in the Netherlands (Erasmus MC, Amsterdam UMC, UMC Utrecht, and Leiden UMC) and their publications. We select the publications of each UMC and ask the experts in these UMCs (which are in most cases the authors of these articles) to judge which annotation method (TERMite or a simple string matching) assigns better disease names to their articles. We then, report the total number of wins of each engine over articles per UMC. The winning engine per article is determined by the experts reflecting that the set of diseases detected by the engine for the article are more relevant to the article compared to the set detected by the other engine.

## Evaluation metrics and results

In this section, we first describe the evaluation metrics used to evaluate the performance of the designed system for annotating articles with rare disease. Then, we present the results and analyze them.

### Evaluation metrics

To evaluate the performance of the proposed approach on annotating documents with rare diseases, we use the datasets described in Sect. 4.1. Since the dataset is compiled from various sources and is not originally designed to evaluate the performance of models on the rare diseases annotation task, it does not cover the majority of the OrphaNet taxonomy. We use common text classification evaluation metrics including Precision, Recall, and F1.

For further analysis, we use the following metrics:

- CorrectRatio: Ratio of samples for which the model detects at least one correct disease.
- ExactRatio: Ratio of samples for which the set of model detection is identical to its ground truth label.

### Results

Table 2 shows the performance of the used approaches in the classification of documents in the test set with OrphaNet concepts. As the results show, the TERMite-based approach achieves a much higher performance than the baseline in this dataset in terms of the evaluation metrics. This method is able to detect at least one of OrphaNet concepts correctly for 79% of the samples in the dataset. Both methods have a high precision but lower recall meaning that they miss to capture some concepts for some documents. A large portions of the missed concepts might be attributed to the inconsistencies in the taxonomy version we used and the version used to annotate the NLM2007, BioASQ5000, and L1000 datasets. Some of the concepts used in these datasets have been deprecated or changed over the years. An example is the following label “*β* thalassemia major” which is changed to “beta-thalassemia”. To reduce the impact of such mismatches, we went through the false negatives and normalized the labels. Another factor that affects recall is the fact that not all synonyms of concepts are included in the taxonomy (especially in the case of acronyms). This can be further addressed by using semantic matching of concepts and documents. However, training such models requires a large-scale dataset with gold labels, which is beyond the scope of this study. The higher precision of the TERMite-based approach might be due to the specificity of the rare diseases which makes it easier for this approach to capture them as this approach is based on the exact and fuzzy matching of the disease names and their synonyms in the text.

**Table 2:** Performance of the TERMite and the baseline on the document classification task. Confidence intervals (95%) are computed using the Wilson score interval

Model	Precision	Recall	F1	CorrectRatio	ExactRatio
Baseline	0.91±0.02	0.41±0.12	0.57±0.02	0.51±0.10	0.32±0.14
TERMite	0.93±0.02	0.73±0.05	0.82±0.03	0.79±0.04	0.59±0.10
TERMite+Baseline	0.92±0.02	0.75±0.04	0.83±0.03	0.81±0.03	0.59±0.10

Table 3 shows the number of wins of each annotation engine over articles published by the four UMCs in the Netherlands in certain diseases in the past five years. The lists of diseases per UMC are included in Table 4.

**Table 3:** Performance of the TERMite and the baseline on the publications of the top four medical centers in the Netherlands

University Medical Center	#wins TERMite	#wins baseline	#ties
Erasmus MC (*N* = 276)	132	15	129
Leiden UMC (*N* = 73)	27	14	35
UMC Utrecht (*N* = 88)	72	2	14
Amsterdam UMC (*N* = 89)	14	26	49

**Table 4:** Top-5 OrphaNet diseases used per UMC for evaluation of the annotation engine

Medical Center	Disease names
Erasmus MC	Tuberous sclerosis complex, Glycogen storage disease due to acid maltase
	deficiency, Glycogen storage disease, Fragile X syndrome,
	Neurofibromatosis type 1
Leiden UMC	Thyroid carcinoma, Acromegaly, Pituitary adenoma, Multiple endocrine
	neoplasia
	type 1, Cushing disease
UMC Utrecht	Lymphoma, Ectopia cordis, Hemophilia A, Hodgkin lymphoma,
	Multiple myeloma
Amsterdam UMC	Niemann-Pick disease type B, Niemann-Pick disease type A, Gaucher disease,
	Pontocerebellar hypoplasia type 8

The results show that overall, the TERMite-based pipeline has a better performance than the string-matching based pipeline and this engine assigns better concepts for the publications of three out of four UMCs. We hypothesize that the difference between performance of methods across different UMCs is due to the difficulty of the surface from of the diseases the UMCs specialize in. For many articles, the concepts assigned by the TERMite and string-matching methods are the same (shown by number of ties). The main reason for this is that rare disease names tend to be very unique and specific which makes them easily detectable by even a simple string-matching approach.

Amsterdam UMC is the only UMC for which the string-matching performs better than the TERMite. The main reason for this is the uniqueness of the diseases in the list of diseases inspected for this UMC. The string-matching which solely works on the exact name can detect such unique diseases with a high precision while the TERMite engine which uses a fuzzy matching approach tries to find mentions similar (but not the same) to the diseases. This process will result in a higher recall, but it can also introduce some noise that can impact the precision negatively. For the other three UMCs and on the non-tie samples, the list assigned by TERMite has significantly higher quality than the list assigned by the string-matching.

**Fig. 3:**
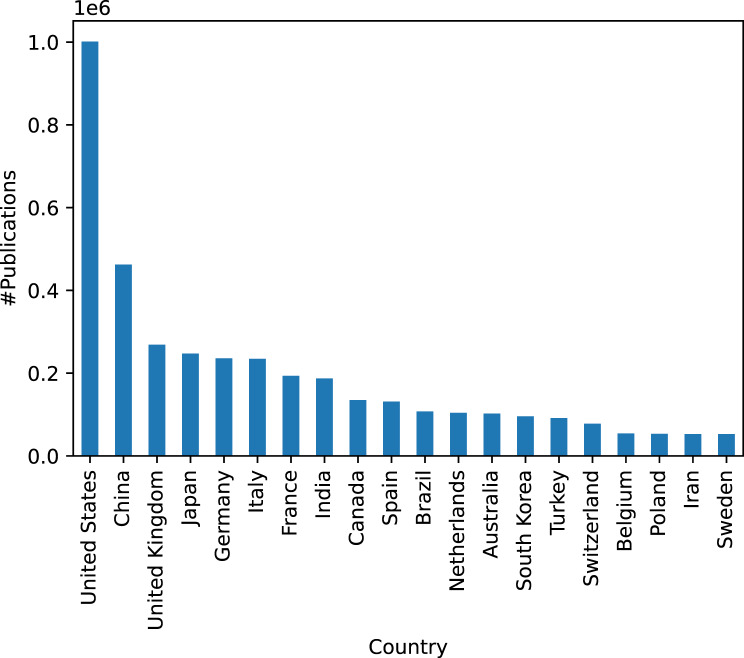
Total number of publications on rare diseases per country. Country-level counts use full counting over author affiliations; all collaborating countries receive a count for a co-authored publication

**Fig. 4:**
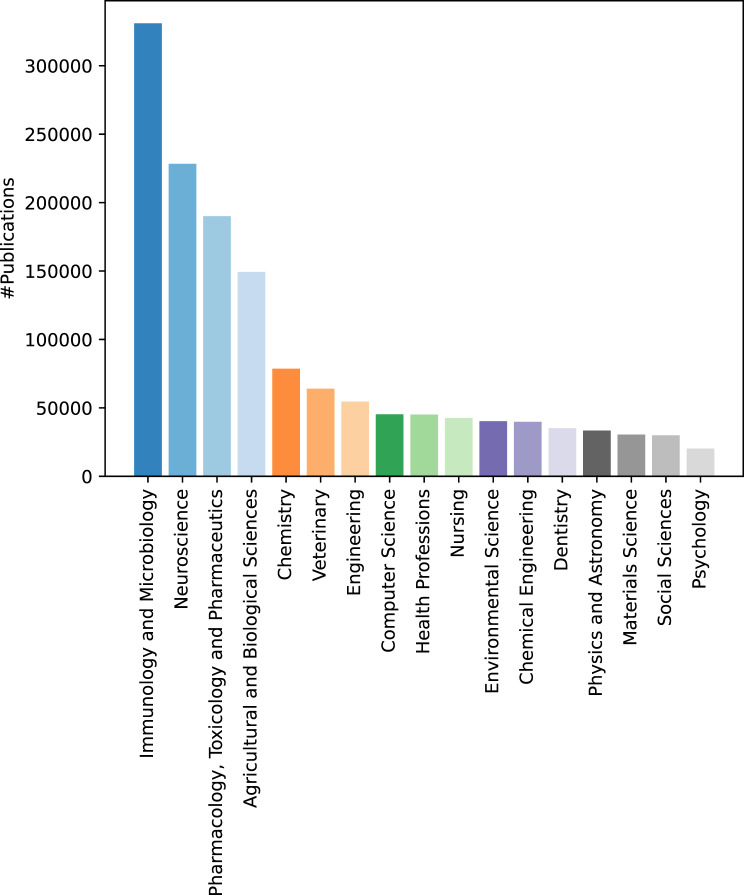
Total number of publications on rare diseases per different subject areas. Subject area level counts use full counting over subject areas; all subject areas of a publication receive a count for the publication. ‘Medicine’ is excluded from list of subject areas. There were  2.8 million publications for ‘Medicine’

**Fig. 5:**
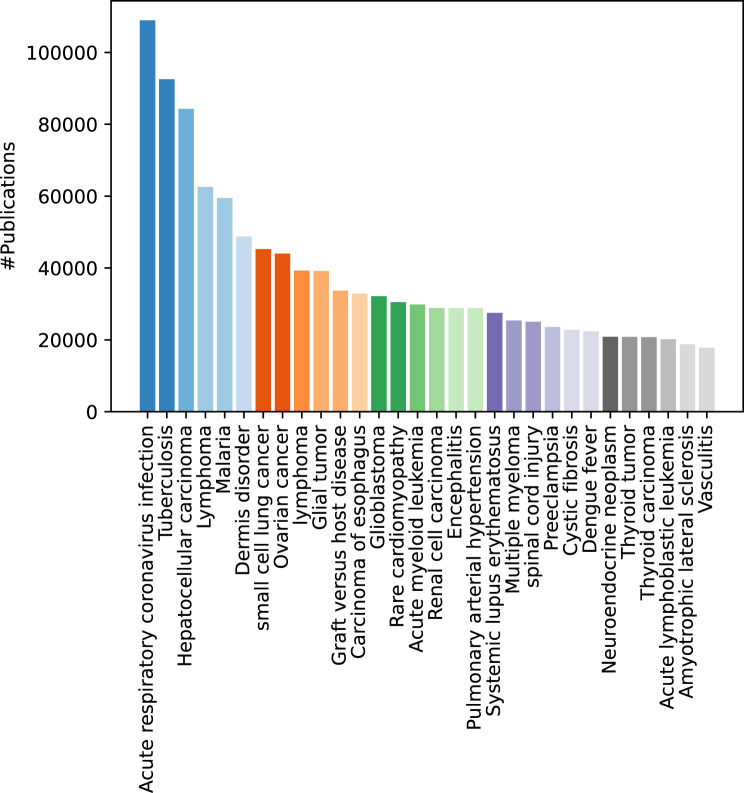
Top 30 diseases with the highest number of publications and the number of publication per each of them

Table 5 shows the overall statistics of the publications on rare diseases (detected by the TERMite-based pipeline) in the past ten years. Overall, there are more than 2.4 million articles detected to be on rare diseases in the past ten years. Figure 3 contains a break down of this number by countries. We only show top ten countries with the highest number of publications. As expected, US has the highest number of publications followed by China. Figure 4 shows the breakdown of publications on rare disease per different science subject areas excluding *Medicine* subject area. These subject areas are extracted from the Scopus database per research article. *Medicine* and relevant fields are the the dominant subject areas as expected. However, domains such as *Engineering* and *Computer Science* also have a relatively high number of publications in the indexed data showing the interdisciplinarity of the research on rare diseases. Figure 5 shows the statistics in terms of number of publications on top 30 diseases with the highest number of publications. As the results show, the *Acute respiratory coronavirus infection* has the highest number of publications followed by *Tuberculosis*.

**Table 5:** Statistics of publications on rare diseases in the 2012–2022 period. Both unique number of publications matched to at least one disease and the total number of matches of diseases to the articles are included

Metric	Number of publications	Number of matches
Matched records for the Netherlands	66,940	104,136
Matched records for EU	647,162	1,048,423
Matched records for the world	2,459,516	3,663,867

### Rare diseases monitor dashboard

**Fig. 6:**
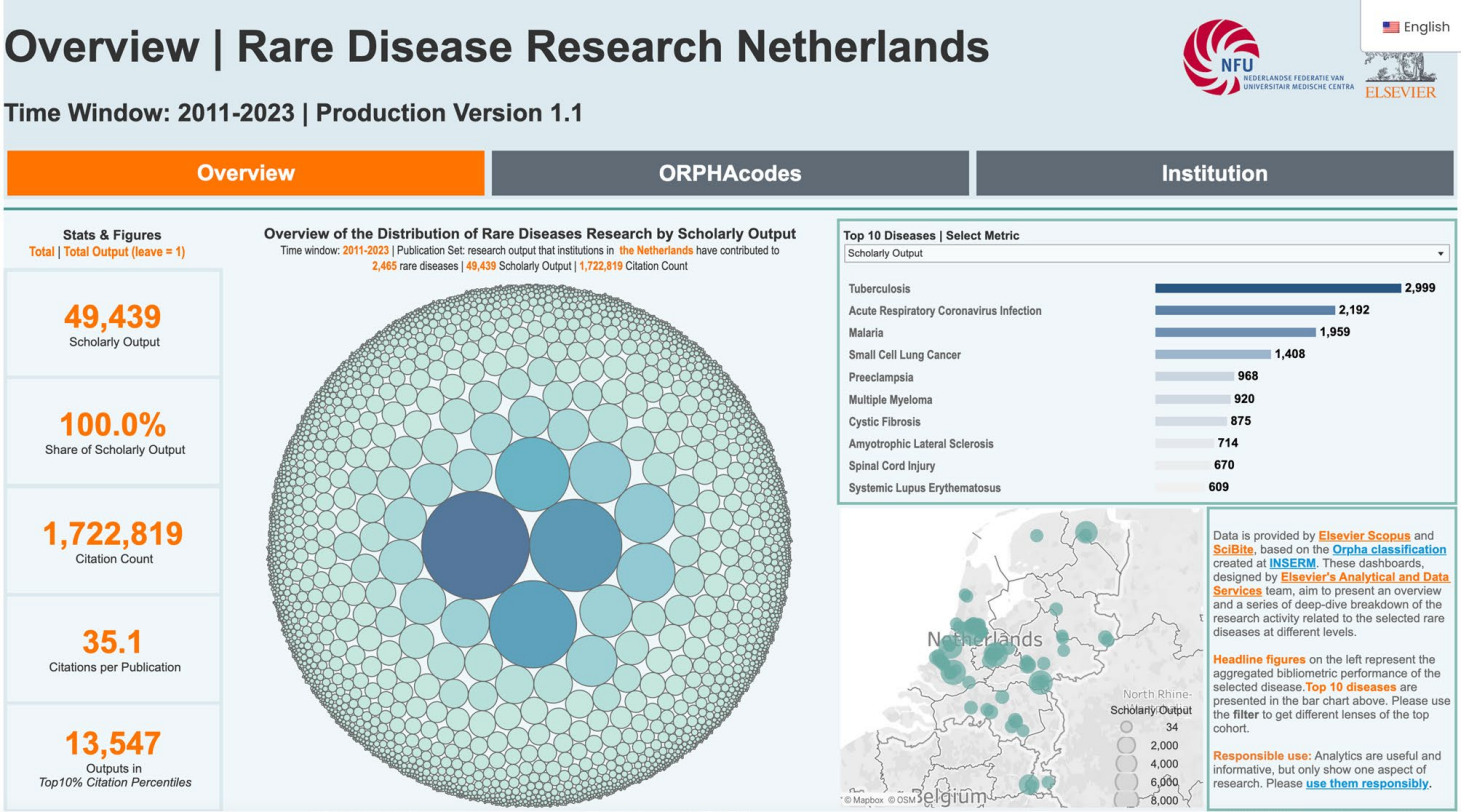
An overview of the rare diseases monitor dashboard

We have built the interactive Rare Diseases Monitor dashboard based on the results of the annotations done by our pipeline. Combined with metadata around articles extracted from the Scopus database, this dashboard shows the publications around different rare diseases. This dashboard facilitates an in-depth investigation of various rare diseases and syndromes through the analysis of peer-reviewed articles, allowing researchers to identify the key institutions contributing to our current understanding of each disorder. Furthermore, the dashboard employs citation analysis to track research impact and knowledge dissemination, aiding in the advancement of knowledge about rare diseases. The dashboard also highlights the organization of information into distinct aggregations representing acknowledged centers of expertise, ERNs, and patient associations. This categorization enables the identification of collaborative opportunities and areas where further collaboration might be fostered. The dashboard offers three distinct views, enabling a comprehensive evaluation of Orpha research at Dutch institutions. The “Overview” section provides a collective assessment of the total number of publications related to rare diseases. In the “ORPHAcodes” section, users can explore research publications associated with specific ORPHAcodes, combinations of ORPHAcodes, and investigate analyses per Expert Center (EC) or ERN. Lastly, the “Institution” section empowers institutions to identify their notable contributions, in terms of publications and citations, to specific rare diseases. Figure 6 shows the overview of the Rare Diseases Monitor dashboard.

Following this study, we conducted an in-depth usability and impact assessment of the Rare Diseases Monitor, which is presented in a separate publication  [35]. That work reports qualitative and quantitative evaluations involving clinicians, researchers, and policymakers, providing further insights into the dashboard’s practical utility and adoption.

## Discussion

Tracking advancement around different rare diseases is of interest for patients suffering from such diseases, researchers and clinicians, as well as the governmental agencies who want to support such advancements. Scientometrical and biliometrical analysis can be seen as one of the means to track such advancements. However, annotating research outputs with rare diseases is not a trivial task for several reseasons including lack of large-enough annotated datasets and challenges associated with usage of taxonomies for such task (such as polysemy, synonymy, and name variations).

In this paper, we addressed some of these challenges by using a highly scalable named entity recognition tool called TERMite. Our automatic data annotation pipeline start with a large collection of articles from one of the largest science databases (Scopus). Each Scopus article is converted to a document by combining the title, abstract, and keywords. Then, the annotation engine uses a set of rule-based as well as fuzzy matching techniques to find mentions of diseases in the documents.

### Key findings

The approach achieves high precision with competitive recall relative to a strong lexical baseline, with improvements attributable to curated normalization and synonym handling. Expert evaluation indicates that the annotations translate to higher perceived concept quality than exact string matching in three of four UMC cohorts, and the resulting semantically indexed corpus enables disease-centric bibliometrics at national and international levels. 

### Biomedical semantics contribution

The work advances representation and evaluation for rare disease semantics by (a) formalizing mappings and normalization for OrphaNet concepts in text and proposing a scaleable pipeline for annotating scientific publications with rare diseases, (b) articulating task-appropriate metrics (Exact/Correct) and evaluation on rare disease annotation, and (c) demonstrating how annotation aggregation supports knowledge organization (by disease, institution, country, and subject area).

### Limitations

A key limitation of this study is the size and coverage of the evaluation dataset. Due to the absence of large-scale, human-annotated corpora specifically aligned with OrphaNet concepts, our ground-truth set was derived from existing MeSH-based datasets. While this approach provides a reproducible benchmarking strategy, it only partially reflects the full OrphaNet taxonomy and therefore may not capture all rare disease concepts present in the literature. We recognize that transformer-based models such as BioBERT have shown strong performance in biomedical named-entity recognition and semantic matching tasks. However, their effective use typically requires substantial amounts of labeled training data, which are currently unavailable for rare disease concepts. Consequently, we focused on unsupervised approaches, including TERMite and string matching, that remain feasible under data-scarce conditions.

### Future work

In this paper, we mainly used unsupervised approaches for indexing articles with rare diseases as there are no large dataset for training supervised models. One possible approach to address this limitation could be using the datasets created for similar taxonomies such as MeSH or ICD10 and then performing a transfer learning to adapt the models trained on those datasets to classify documents with OrphaNet concepts. We did not use the full text of articles to assign OrphaNet concepts due to the noise that leads to a lot of false positives. More research needs to be done on possible approaches to either prevent noisy annotations in the annotation stage or post-process the annotations and remove falsely assigned concepts to the articles. We have built the *Rare Diseases Monitor* dashboard based on the outcomes of the annotation pipeline introduced in this paper. We would like to perform user studies to understand the utility of such annotations for the users and their possible applications. There remains significant potential for further development. Establishing a Europe-wide dashboard would be highly valuable in facilitating more efficient ERN membership processes and fostering research collaboration. It could also provide crucial insights for patients who currently lack access to appropriate treatment in their own countries due to limited local expertise. Beyond this, there are additional opportunities to anticipate future needs and meaningfully extend the scope of the project.

## Conclusion

We deliver a semantically indexed view of rare disease research by operationalizing the OrphaNet taxonomy for large-scale literature annotation and evaluation. The resulting corpus and dashboard support disease-centric discovery and monitoring for clinicians, researchers, and policymakers. While recall is constrained by label drift and abstract-only text, our approach establishes a reproducible semantic framework that can be extended with embedding-based matching, additional data sources, and broader geographic coverage.

To evaluate the performance of our pipeline we compiled a ground-truth dataset by combining a set of existing annotated datasets (which are mainly annotated by MeSH terms). Our evaluation on this dataset shows that the proposed approach has a very high precision but a bit lower recall. The high precision can be attributed to the fact that rare diseases are very specific and since TERMite is mainly a rule-based system, the matches it finds are very accurate. On the other hand, the lower recall might be due to some changes in the taxonomy as well as the fact that the rule-based system might miss some variations in the disease names. We further evaluated the quality of the annotations done by the pipeline by conducting a user study on top four medical centers in the Netherlands. Based on the user evaluations, the OrphaNet concepts assigned to the articles published by these medical centers have a high quality. The resulting annotated article collection contains over 2.4 million articles annotated at least by one rare disease.

## Data Availability

No datasets were generated or analysed during the current study.

## Abbreviations

ERN: European Reference Network
NLP: Natural Language Processing
UMC: University Medical Center
EC: Expert Center

## Footnotes

1 https://www.rijksoverheid.nl/onderwerpen/kwaliteit-van-de-zorg/expertisecentra-zeldzame-aandoeningen

2 https://health.ec.europa.eu/european-reference-networks_en

3 http://scopus.com/

4 https://scibite.com/platform/termite/

5 https://public.tableau.com/app/profile/dennis.yu4874/viz/Rare_diseses_dashboard_DraftVersion0_1/Front

6 https://lincsportal.ccs.miami.edu/datasets/view/LDS-1293
